# Genetic and biological characterisation of three cryptic *Eimeria* operational taxonomic units that infect chickens (*Gallus gallus domesticus*)^☆^

**DOI:** 10.1016/j.ijpara.2020.12.004

**Published:** 2021-07

**Authors:** Damer P. Blake, Vladimir Vrba, Dong Xia, Isa Danladi Jatau, Simon Spiro, Matthew J. Nolan, Greg Underwood, Fiona M. Tomley

**Affiliations:** a Pathobiology and Population Sciences, Royal Veterinary College, Hawkshead Lane, North Mymms AL9 7TA, UK; b Bioproperties Pty Ltd, 36 Charter St, Ringwood, Victoria 3134, Australia; c Department of Parasitology and Entomology, Faculty of Veterinary Medicine, Ahmadu Bello University, Zaria, Nigeria; d Wildlife Health Services, Zoological Society of London, Regent’s Park, London NW1 4RY, UK

**Keywords:** Chickens, *Eimeria*, Cryptic species, Operational taxonomic unit, Genome sequencing, Food security

## Abstract

More than 68 billion chickens were produced globally in 2018, emphasising their major contribution to the production of protein for human consumption and the importance of their pathogens. Protozoan *Eimeria* spp. are the most economically significant parasites of chickens, incurring global costs of more than UK £10.4 billion per annum. Seven *Eimeria* spp. have long been recognised to infect chickens, with three additional cryptic operational taxonomic units (OTUs) first described more than 10 years ago. As the world’s farmers attempt to reduce reliance on routine use of antimicrobials in livestock production, replacing drugs that target a wide range of microbes with precise species- and sometimes strain-specific vaccines, the breakthrough of cryptic genetic types can pose serious problems. Consideration of biological characteristics including oocyst morphology, pathology caused during infection and pre-patent periods, combined with gene-coding sequences predicted from draft genome sequence assemblies, suggest that all three of these cryptic *Eimeria* OTUs possess sufficient genetic and biological diversity to be considered as new and distinct species. The ability of these OTUs to compromise chicken bodyweight gain and escape immunity induced by current commercially available anticoccidial vaccines indicates that they could pose a notable threat to chicken health, welfare, and productivity. We suggest the names *Eimeria lata* n. sp., *Eimeria nagambie* n. sp. and *Eimeria zaria* n. sp. for OTUs x, y and z, respectively, reflecting their appearance (x) or the origins of the first isolates of these novel species (y, z).

## Highlights

• Biology and genetics suggest cryptic *Eimeria* Operational Taxonomic Units (OTUs) from chickens are new species.
• New *Eimeria* spp. that infect chickens are pathogenic and require control.
• Anticoccidial vaccination of chickens does not control three new *Eimeria* spp.

## Graphical abstract

**Figure:**
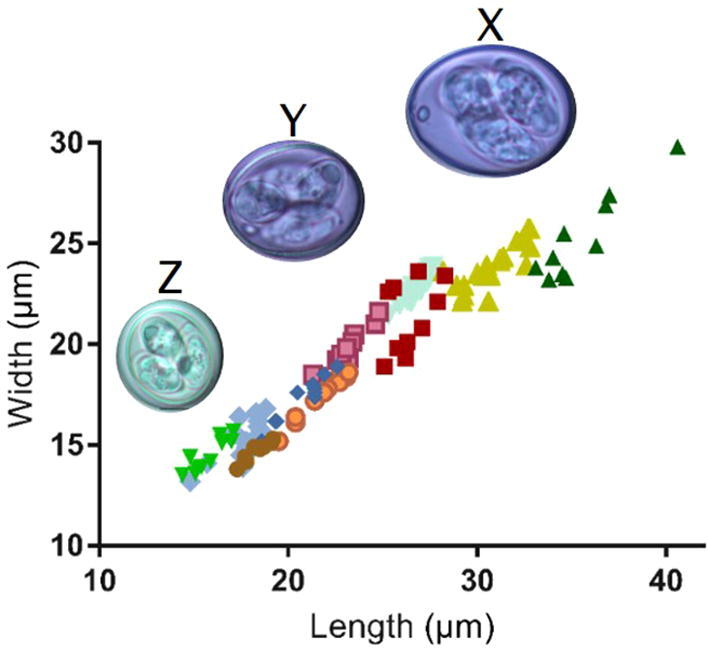

## 1 Introduction

*Eimeria* are apicomplexan parasites that can cause the disease coccidiosis, most notably in farmed animals such as chickens where the global cost of coccidiosis is estimated to exceed UK £10.4 billion per annum (USD $14.4 billion; (Blake et al., 2020)). The consequences of infection in chickens can include haemorrhagic enteritis, most commonly caused by *Eimeria tenella* but also by *Eimeria necatrix* and *Eimeria brunetti*, or malabsorption, frequently associated with *Eimeria acervulina* or *Eimeria maxima*, or to a lesser extent with *Eimeria mitis* and *Eimeria praecox* (Reid et al., 2014). Reports of additional ‘species’ in chickens such as *Eimeria hagani* or *Eimeria mivati* have appeared sporadically but then found to be *nomina dubia* (Edgar and Seibold, 1964, Shirley et al., 1983, Chapman, 2003, Vrba et al., 2011). More recent sequence-led studies identified three cryptic *Eimeria* genotypes circulating in Australian chicken populations that appeared distinct from the recognised species and which to date have been considered as novel operational taxonomic units (OTUs). First identified by capillary electrophoretic resolution of internal transcribed spacer (ITS)-2 PCR amplicons (Morris et al., 2007), subsequent deep-amplicon sequencing of more isolates revealed 10 sequence types that resolved into three distinct phylogenetic clusters, termed OTUx, y, and z (Cantacessi et al., 2008). Occurrence of these three OTU genotypes was initially thought to be restricted to the southern hemisphere (Clark et al., 2016, Jatau et al., 2016), but recent next-generation ITS-2 amplicon sequencing has detected all three in North America (Hauck et al., 2019). While short amplicon sequence-based surveillance is informative, it is difficult to assess the true provenance of candidate new *Eimeria* spp. without robust morphometric and pathological data derived from pure isolates, as well as much more detailed molecular genomic descriptions.

*Eimeria* spp. that infect chickens are primarily controlled by drug- or vaccine-mediated prophylaxis. Anticoccidial chemoprophylaxis is most common, using a range of ionophore and/or chemical products delivered by routine dietary supplementation to control, but not eradicate, *Eimeria* (Chapman, 1999). Most registered anticoccidial drugs are relatively broad spectrum, killing or inhibiting all seven of the recognised *Eimeria* spp. (Chapman, 1997). It is highly likely that these drugs are similarly effective against cryptic *Eimeria* OTUs, with the likelihood of OTUs acquiring genetic resistance to drugs, also similar to the existing well-known parasites. In contrast, vaccination is achieved by direct or indirect oral inoculation of varied formulations of live wild-type or attenuated *Eimeria* spp. (Shirley et al., 2005) and immunity acquired following infection is highly species-, and in some examples, strain-specific (McDonald and Shirley, 2009). This exquisite specificity of immunoprophylaxis means that coccidiosis vaccines may be ineffective when chickens are infected by antigenically distinct parasites, for example the cryptic OTU genotypes. While these differences in specificities of anticoccidial drugs and vaccines have long been acknowledged and managed, the balance in the field is now changing. Resistance to anticoccidial drugs develops rapidly and has become widespread, requiring regular monitoring for efficacy and/or cycling between different products (Peek and Landman, 2011). Furthermore, public and legislative pressure to reduce the use of antimicrobial drugs in livestock production is promoting increased uptake of anticoccidial vaccines, most notably in the United States where more than 30% of commercial broiler chickens sold since 2016 received an anticoccidial vaccine (Blake et al., 2020). The consequences of changing the balance of control from broad-spectrum anticoccidial drugs to narrow-spectrum species-specific vaccines on field parasite populations are not yet clear. The presence of undefined *Eimeria* OTU genotypes exacerbates the problem. It is now timely to define in much more detail *Eimeria* OTUs x, y, and z, exploring their biological traits, genetic and antigenic diversity, and comparing these directly with the seven recognised *Eimeria* spp. that infect domestic chickens.

## 2 Materials and methods

### 2.1 Parasite collection

In Australia, oocysts representing OTUx and y genotypes were isolated from a commercial broiler-breeder farm (Nagambie, Victoria) that repeatedly suffered increased chicken morbidity and mortality at 5–8 weeks of age with the appearance of coccidiosis, despite prior anticoccidial vaccination (Morris et al., 2007). Oocysts corresponding to OTUz were subsequently isolated from the same farm during routine testing for *Eimeria* spp. and identified using a capillary electrophoresis technique (Cantacessi et al., 2008).

In Nigeria, faecal samples were collected from commercial broiler chicken farms in Kaduna state and screened using OTU-specific PCR for the presence of OTUx, y, or z genotype oocysts, as described previously (Jatau et al., 2016). Samples found to contain one or more of the target genotypes were first amplified by in vivo passage followed by faecal harvest. Oocysts of the target genotypes were subsequently selected by in vivo passage using Paracox® 8 vaccinated chickens (as described in section 2.2.2).

### 2.2 Parasite amplification, selection and maintenance

#### 2.2.1 Routine parasite amplification

Oocysts were propagated through specific pathogen-free (SPF) 28-day-old Lohmann Valo chickens. SPF chickens were purchased from the Animal & Plant Health Agency, Weybridge, UK. Oocysts were maintained, prepared and administered as described elsewhere (Eckert et al., 1995). Total daily faecal output was collected for oocyst recovery following a standard saturated saline flotation protocol (Eckert et al., 1995) from groups of two to 10 chickens accommodated on wire floors, following UK Home Office regulations. Initially, faeces were collected and processed daily from day 4 to day 9p.i. to ensure recovery of the majority of excreted oocysts from the unknown species/OTUs. Subsequently, faeces were collected and pooled for harvest over the first 72 h of patent oocyst excretion once the pre-patent period had been established for each isolate. Oocysts were sporulated in 2% (w/v) potassium dichromate at room temperature for ~90 h to ensure maximum sporulation of each uncharacterised oocyst population, then stored at +4 °C and used within 3 months of harvest.

#### 2.2.2 Selective isolation and amplification of OTU genotype oocysts

Australian OTUx, y, and z isolates were repeatedly passaged through multi-vaccinated chickens to obtain monospecific preparations. Chickens were vaccinated using Eimeriavax 4 m, supplemented by the additional species *E. mitis* and *E. brunetti*, as well as other purified OTUs once they became available. To further purify the OTU isolates, oocysts were diluted and in vivo infections initiated with ~12 oocysts per bird. Successful purification of each OTU from the recognised species was confirmed with the same method as used for species detection (ITS-2 capillary electrophoresis, (Morris et al., 2007)). OTUx and z isolates were stored as sporocyst stocks in liquid nitrogen, but a viable OTUy isolate has been lost and only extracted genomic DNA was available.

Nigerian field oocyst populations found by PCR to contain one or more OTU genotypes were passaged through pairs of Paracox® 8 (MSD Animal Health, Milton Keynes, UK) triple vaccinated Lohmann Valo chickens. Briefly, each chick received 1 ml of 1× Paracox® 8 by oral gavage at 21 days of age. Subsequently, 1 ml of 5× Paracox® 8 was administered at 28 days of age, followed by 1 ml of 10× Paracox® 8 at 32 days of age. Chickens were challenged by oral gavage using candidate field oocyst populations 15 days later (47 days of age). Field populations prior to selection are identified by the suffix [1], indicating generation 1. Progeny oocysts, indicated by the suffix [2], were recovered and processed as described above. A vaccinated, 10× Paracox® 8 challenge group was included in every selective passage as a control for the species-specific immune selective barrier. An unvaccinated, 10× Paracox® 8 challenged group was included as a control for vaccine viability. The presence/absence of each *Eimeria* sp./OTU genotype was assessed in the parasite populations used for dosing and after in vivo passage by PCR, indicating (i) parasite escape from anticoccidial vaccination and (ii) the identity of apparently pure OTU genotype populations (i.e. the absence of other *Eimeria* spp./OTU genotypes, within the limit of PCR sensitivity). A viable, pure line of Nigerian OTUy parasites is not currently available.

### 2.3 Eimeria species and OTU genotype specific PCR

Total genomic DNA was extracted from purified *Eimeria* oocysts using a QIAamp Blood and Tissue DNA mini kit (Qiagen, Hilden, Germany) including a preliminary physical disruption step using a Mini Beadbeater-8 or -24 (Biospec Products, Bartlesville, USA) as described previously (Jatau et al., 2016). The presence of *E. acervulina*, *E. brunetti*, *E. maxima*, *E. mitis*, *E. necatrix*, *E. praecox*, and/or *Eimeria tenella* was determined by standard PCR using primers developed previously for *Eimeria* spp.-specific quantitative PCR and validated with a panel of samples representative of Africa, Europe, North and South America (Vrba et al., 2010, Fornace et al., 2013). Briefly, each reaction contained 25–100 ng of template genomic DNA in 1 µl, 20 pmol forward and reverse primers (Supplementary Table S1), 1× MyTaq premix (Bioline, London, UK), made up to a final volume of 25 µl with molecular grade water (Sigma-Aldrich, St Louis, USA). Standard cycle parameters were initial denaturation: 1 × 5 min at 94 °C, followed by 30 cycles of denaturation: 0.5 min at 94 °C, annealing: 0.5 min at variable ^o^C (Supplementary Table S1), and extension: 1 min at 72 °C, completed by final extension: 1 × 10 min at 72 °C. Expected amplicon sizes were as shown in Supplementary Table S1. All candidate OTU amplicons representing new *Eimeria* field populations were prepared for confirmation by direct Sanger sequencing (GATC Biotech, Konstanz, Germany) using the same primers as employed in the original amplification. Sequence data were visualised and curated using CLC Main Workbench version 8.0.1 (Qiagen).

### 2.4 Oocyst morphology

Sporulated oocysts were photographed and measured under oil immersion at ×100/×10 magnification using an Olympus DP20 camera mounted on a CX41 microscope (Olympus, Tokyo, Japan) and the program ImageJ, version 1.51 (Schneider et al., 2012). All measurements described here are given in µm, presenting the mean followed by the range in parentheses. Structural characteristics of the oocysts described here have been abbreviated as recommended elsewhere (Wilber et al., 1998): oocyst wall (OW), oocyst length (L), oocyst width (W), oocyst length:width ratio (L/W), micropyle (M), oocyst residuum (OR), polar granule (PG), sporocyst residuum (SR), Stieda body (SB) and refractile body (RB).

### 2.5 In vivo characterisation

#### 2.5.1 Pre-patent period and fecundity

Initially, two 28-day old SPF Lohmann Valo chickens, accommodated in one cage, were inoculated with 100 sporulated OTUz (Aus) oocysts by oral gavage. For a preliminary estimate of the pre-patent period all excreted faecal material was collected daily, from days 3–9p.i., and assessed for the presence of OTUz oocysts by faecal flotation in saturated saline solution as described previously (Blake et al., 2005). Oocysts were first noted 6 days p.i. Relative fecundity was assessed by comparison of the total OTU oocyst output per chicken with expected output for the seven recognised species (Bumstead and Millard, 1992). Subsequently, eight 28-day old SPF Lohmann Valo chickens were accommodated in single bird cages and inoculated with 30,000 (*n* = 3), 3000 (*n* = 3) or 0 (mock infected, *n* = 2) sporulated OTUz (Aus) oocysts by oral gavage. All excreted faecal material was collected in 5 h windows, starting from 120 h p.i., to detect first OTUz oocyst excretion and determine the pre-patent period.

The process was repeated for OTUx (Aus) with the following minor alterations. For identification of the pre-patent period individually caged chickens were inoculated with 10,000 (*n* = 3), 1000 (*n* = 3) or 0 (mock infected, *n* = 2) sporulated OTUx (Aus) oocysts by oral gavage. Faecal collection started from 115 h p.i., followed by three 5 h windows.

#### 2.5.2 Intestinal site(s) of replication

The enteric location of intracellular OTU parasites was assessed 112 h after oral inoculation by standard PCR using total genomic DNA extracted from six locations within the chicken gastrointestinal tract. Pairs of Lohmann Valo chickens kept in separate cages were inoculated with 3000 OTUz (Aus) oocysts, 1000 OTUx (Aus) oocysts, or mock inoculated (negative control) at 28 days of age. Immediately post-mortem, ~5 cm long intestinal sections were collected from (1) the mid-point of the duodenum, (2) the distal end of the duodenum, (3) between the jejunum and ileum (around Meckel’s diverticulum), (4) the mid-point of the ileum, (5) the mid-point of one caecal pouch, and (6) the rectum. Each section was dissected, opened longitudinally, washed twice in clean 1× PBS (pH 7.4; Fisher Scientific, Loughborough, UK) and preserved in RNAlater as recommended by the manufacturer (Fisher Scientific). Subsequently, total genomic DNA was extracted from each ~5 cm section using a QIAamp Blood and Tissue DNA mini kit (Qiagen) as described previously (Nolan et al., 2015). Genotype-specific PCR targeting OTUz or OTUx was used to determine the range of parasite occurrence in each sampled chicken using 1 µl of genomic DNA from each intestinal location, as described in section 2.3. Mock infected and no template samples were used to provide a negative control.

#### 2.5.3 Pathogenicity: influence of infection on bodyweight gain

Commercial Ross 308 broiler chickens (PD Hook, Bampton, UK) were used to assess the consequences of OTUz or OTUx infection on bodyweight gain (BWG) under simulated commercial conditions. In consecutive studies, Ross 308 chicks were received on day of hatch and accommodated in adjustable circular wire pens on the floor with clean wood shavings at half the recommended commercial stocking density, equivalent to 5.38 chickens per square metre (Aviagen, 2014; http://goldenpoultry.com/wp-content/uploads/2014/09/Ross-Broiler-Handbook-2014i-EN.pdf). In the first study, three groups of eight 21-day old Ross 308 chicks were weighed and inoculated with a mock (Group 1, no oocysts), low (Group 2, 5000 OTUz (Aus) oocysts) or high (Group 3, 100,000 OTUz (Aus) oocysts) oral parasite challenge. Each chicken was weighed again 10 days post challenge (31 days old) to assess the impact of infection on BWG. A second study followed the same protocol, with the exception that Group 2 was inoculated with 1000 OTUx (Aus) oocysts, and Group 3 received 10,000 OTUx (Aus) oocysts.

### 2.6 Vaccination and challenge studies

Preliminary data describing the escape and replication of Nigerian OTUx, y, and z genotype parasites in Paracox® 8 vaccinated chickens were collected during selective isolation and amplification, as described above (Table 1, Trial 1). Subsequently, one Nigerian parasite population recovered after selective passage was used in a quantitative vaccination/challenge study (Table 1, Trial 2). Groups of six SPF Lohmann Valo chickens were left unvaccinated (Groups 1 and 3) or Paracox® 8 vaccinated using the triple inoculation protocol described in section 2.2.2 (Groups 2 and 4). At 47 days of age chickens from each group were relocated into single bird cages and challenged by oral gavage using 1 ml of 10× Paracox-8 (Groups 1 and 2) or 250 sporulated oocysts of the selected Nigerian OTU *Eimeria* population (Groups 3 and 4). Total daily oocyst output was determined per chicken using saturated saline flotation, as described above, from days 4–8 post challenge.

**Table 1:** Summary of in vivo vaccination studies demonstrating Operational Taxonomic Unit (OTU) genotypes escape from commercial live anticoccidial vaccines.

Trial	Vaccination	Challenge^1^	No.	Log_10_ oocysts per chicken (+/− SEM)	Present (PCR confirmation)
1	None	Paracox® 8	2	5.08 (na)	
	Paracox® 8 (x3)	Paracox® 8	2	Nd	
	Paracox® 8 (x3)	Ng1[1]: A,N,T,x,y	2	7.44 (na)	x, y
	Paracox® 8 (x3)	Ng2[1]: A,Ma,Mi,T,x,y,z	2	7.49 (na)	A, x, y, z
	Paracox® 8 (x3)	Ng3[1]: A,B,Mi,N,T,x,y	2	6.3 (na)	x, y
2	None	Paracox® 8	6	5.38 (0.43)^a^	
	Paracox® 8 (x3)	Paracox® 8	6	nd^b^	
	None	Ng1[2]: x,y	6	7.47 (0.35)^c^	x, y
	Paracox® 8 (x3)	Ng1[2]: x,y	6	7.32 (0.32)^c^	x, y
3	None	Paracox® 8	5	5.32 (0.38)^a^	
	Paracox® 8 (x3)	Paracox® 8	5	nd^b^	
	None	x (Nig)	5	7.14 (0.42)^c^	x
	Paracox® 8 (x3)	x (Nig)	5	7.11 (0.60)^c^	x
	X	x (Nig)	5	nd^b^	
	None	z (Nig)	5	7.48 (0.65)^c^	z
	Paracox® 8 (x3)	z (Nig)	5	7.53 (0.58)^c^	z
	Z	z (Nig)	5	nd^b^	
4	None	z (Aus)	10	6.71 (na)	z
	MMAT+NB	z (Aus)	10	6.64 (na)	z
	Z	z (Aus)	10	Nd	

na, not applicable; nd, none detected; A, *Eimeria acervulina*; B, *Eimeria brunetti*; Ma, *Eimeria maxima*; Mi, *Eimeria mitis*; N, *Eimeria necatrix*; P, *Eimeria praecox* (not represented); T, *Eimeria tenella*; x, OTUx; y, OTUy; z, OTUz.

Different superscript letters within a trial indicate statistically significant differences (*P* < 0.05).

1 *Eimeria* spp. and genotypes present in the starting dose are indicated.

The ability of OTUx and OTUz to escape immunity induced by a live anticoccidial vaccine was quantified using Paracox® 8, including eight groups of five Ross 308 broiler chickens (Table 1, Trial 3). Groups 1, 3, and 6 were left unvaccinated, Groups 2, 4, and 7 were vaccinated using a single oral Paracox® 8 dose, Group 5 was vaccinated by oral gavage using 1000 OTUx (Nig) oocysts in 0.25 ml, and Group 8 was vaccinated by oral gavage using 1000 OTUz (Nig) oocysts in 0.25 ml. All vaccinations were administered at 1 day of age. The success of vaccination was confirmed by the presence/absence of excreted oocysts seven and 14 days post-vaccination using saturated saline flotation. Subsequently, chickens in Groups 1 and 2 were challenged by oral gavage using 10× Paracox® 8, demonstrating vaccine viability and efficacy. Groups 3, 4, and 5 were challenged using 1000 OTUx (Nig) oocysts, demonstrating viability of the challenge line and capacity for escape from heterologous or homologous vaccination. Groups 6, 7, and 8 were challenged using 1000 OTUz (Nig) oocysts, demonstrating viability of the challenge line and capacity for escape from heterologous or homologous vaccination. All challenge doses were administered at 21 days of age. Total oocyst excretion was calculated per chicken for each group from 4 to 8 days p.i.

Finally, to assess vaccine escape for OTUz (Aus) from immunity induced following vaccination with an Australian-derived vaccine, a small-scale study was completed using three groups of 10 SPF AusSPF, layer-type chickens (Table 1, Trial 4). Chickens in Group 1 were left unvaccinated (control). Chickens in Group 2 were vaccinated using the parasite lines included in HuveGuard® MMAT+NB once, by eye-drop at 1 day of age (heterologous vaccine/challenge). Note, HuveGuard® MMAT+NB (Huvepharma, Antwerp, Belgium) contains attenuated isolates of *E. acervulina*, *E. brunetti*, *E. maxima*, *E. mitis*, *E. necatrix*, and *E. tenella*, but not *E. praecox*. Chickens in Group 3 were vaccinated using 1000 OTUz (Aus) oocysts by eye-drop at 1 day of age (homologous vaccine/challenge). The success of vaccination was confirmed by the presence/absence of excreted oocysts seven, 14 and 21 days post-vaccination. Subsequently, chickens in all three groups were challenged by oral gavage of 20,000 OTUz (Aus) oocysts at 21 days of age. Chickens in each group were kept together, so statistical analysis was not possible. Total oocyst excretion was calculated for each group from 4 to 8 days p.i. and presented as total oocyst output per chicken.

### 2.7 Genome sequencing and analyses

#### 2.7.1 Nucleic acid preparation and sequencing

Total genomic DNA was extracted from purified Australian OTUx, y, and z oocysts as described for PCR. The absence of other contaminating *Eimeria* spp. or OTU genotypes was confirmed by PCR. Quantity and quality of the genomic DNA was assessed by NanoDrop and Qubit high sensitivity kit (Thermo Scientific, Massachusetts, United States). Purity was assessed using *Eimeria* spp.- and genotype-specific PCR. Samples were diluted to 0.2 ng/µl (1 ng total) of genomic DNA and used to prepare ~1 Kb insert Nextera XT DNA sequencing libraries including 12 rounds of PCR. Indexed libraries were pooled and sequenced using an Illumina MiSeq (Source BioScience, Nottingham, UK), producing 300 bp paired end reads. Sequences were quality controlled, adapters removed and assembled without a reference using CLC Assembly Cell software at Source BioScience, creating preliminary genome sequence assemblies for each OTU.

#### 2.7.2 Phylogenetic analyses

Draft genome sequence assemblies for OTUx, y, and z were used to identify 18S rDNA and mitochondrial cytochrome C oxidase I (COI or *coxI*) sequences for phylogenetic analysis using local BLAST in CLC Main Workbench (version 8.0.1) with default parameters. Additionally, fragments of the 18S rDNA and COI loci were amplified from genomic DNA representing Australian and Nigerian OTUx, y, and z parasites as described elsewhere (Schwarz et al., 2009). Sequences are available from the European Nucleotide Archive under the accession number **PRJEB23613**. Amplicons of the anticipated sizes were sequenced using the same primers (GATC Biotech) and assembled using CLC Main Workbench version 8.0.1. Reference 18S rDNA sequences were identified for the seven *Eimeria* spp. that infect chickens using the SILVA ribosomal RNA database (Quast et al., 2013), selecting up to five high quality sequences per species, one sequence per published study where possible. Reference COI sequences were identified for the same seven *Eimeria* spp. using PubMed/Nucleotide (https://www.ncbi.nlm.nih.gov/nucleotide/), again selecting up to five high quality sequences per species, one sequence per published study where possible. Sequences in the 18S rDNA and COI datasets were aligned using CLC Main Workbench with the ‘very accurate’ (slow) option and default parameters, then exported to MEGAX (Kumar et al., 2018). Optimal phylogenetic models were identified using Akaike's Information Criterion (AIC). The Maximum Likelihood (ML), Neighbour Joining (NJ), and Maximum Parsimony (MP) methods were used to estimate sequence phylogeny, all with 1000 bootstrap iterations. The optimal trees inferred using NJ with the Kimura 2-parameter method are presented unless stated otherwise. Pairwise genetic distances were calculated using the Maximum Composite Likelihood model and used to identify (i) the maximum distance within recognised species/OTU groups, (ii) the minimum distance between recognised species/OTU groups, and (iii) the fold difference between the two.

To improve genome coverage in the phylogenetic analyses, gene models were predicted for all three OTU genomes using the ab initio program Augustus v.3.3 (Stanke et al., 2006). For each OTU genome, Augustus was trained with genome annotations retrieved from ToxoDB (release 30), where *E. maxima* models were used for OTUx, *E. brunetti* models for OTUy, and *E. acervulina* models for OTUz. *Eimeria* genome sequence assemblies for the *E. acervulina*, *E. brunetti*, *E. mitis*, *E. necatrix*, *E. praecox*, and *E. tenella* Houghton strains, and the *E. maxima* Weybridge strain, were accessed through ToxoDB (Gajria et al., 2008, Reid et al., 2014). The genome assembly for *Eimeria falciformis* was accessed to provide an outgroup (Heitlinger et al., 2014). Orthologous coding sequences within all eight reference *Eimeria* genomes and all three draft OTU genomes were identified and clustered to ortholog groups by OrthoMCL v2.0 (Li et al., 2003). Where complete sets of orthologous amino acid sequences were identified for each *Eimeria* sp./genotype they were aligned using MAFFT v7 (Katoh and Standley, 2013). Highly variable sites were trimmed using Trimal (Capella-Gutierrez et al., 2009) and amino acid sequence alignments were then concatenated using FASconCAT (Kuck and Meusemann, 2010), prior to phylogenetic analysis. The optimal phylogenetic model was identified using MEGAX, before ML and NJ phylogenies were created as before. Additionally, a Bayesian inference analysis was undertaken using MrBayes through the Topali interface (Huelsenbeck and Ronquist, 2001, Milne et al., 2009). The General Time Reversible model was used with a gamma distribution (GTR+G), with four runs, 1,000,000 generations, a sample frequency of 10% and 25% burn-in.

### 2.8 Statistical analysis

All statistical analyses were undertaken with IBM SPSS Statistics (version 26), using ANOVA followed by a post-hoc least significance difference (LSD) test where *P* < 0.05 was considered to be significant. Graphs were prepared using GraphPad Prism (version 8.0.0).

### 2.9 Ethical review

The studies described here were carried out in strict accordance with the Animals (Scientific Procedures) Act 1986, an Act of Parliament of the United Kingdom. All sample collection, animal studies, and protocols were approved by the Royal Veterinary College (UK) Animal Welfare and Ethical Review Board (AWERB) and the United Kingdom Government Home Office, the Bioproperties Animal Ethics Committee (Glenorie, NSW, Australia), and/or the Animal Ethics Committee of Ahmadu Bello University (Nigeria). *Eimeria* samples were imported into the UK under an Import of Animal Pathogens Order (IAPO) permit issued by the Animal & Plant Health Agency, UK.

## 3 Results

### 3.1 Oocyst morphology for OTUs x, y, and z

The type host for the oocysts used and described here is the domestic chicken, *Gallus gallus domesticus* (Linnaeus, 1758); defined by the Taxon Version Key NHMSYS0020975169 and the NCBI taxon code txid9031. Phototypes of sporulated oocysts of the OTUx, y, and z genotypes have been deposited in the Pathology Museum of the Royal Veterinary College, University of London, UK, as type specimens (reference RVC_I_073), as recommended (Bandoni and Duszynski, 1988). OTUs x, y, and z have been assigned NCBI taxon codes 2052603, 2052604, and 2052605, respectively.

**Fig. 1:**
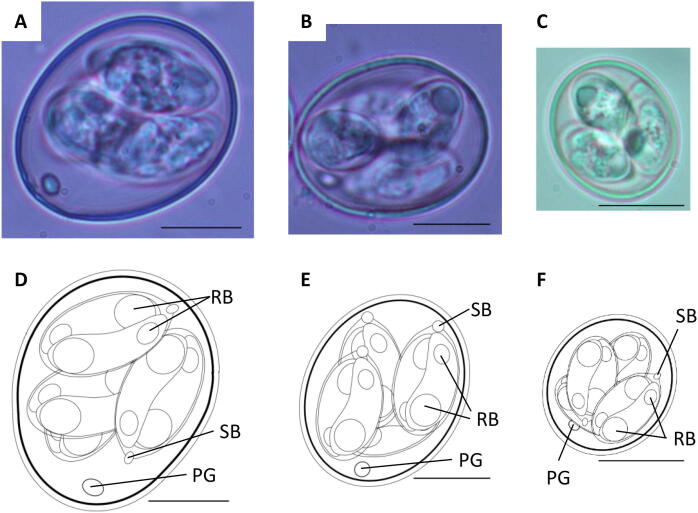
Sporulated oocysts of the *Eimeria* Operational Taxonomic Unit (OTU) genotypes x, y, and z collected from domestic chickens (*Gallus gallus domesticus*). Photomicrographs of sporulated oocysts are shown for (A) OTUx, (B) OTUy and (C) OTUz. Composite line drawings are shown for (D) OTUx, (E) OTUy and (F) OTUz. RB, residual body; SB, stieda body; PG, polar granule. Scale bars = 10 µm.

Following sporulation, oocysts of the OTUx, y, and z genotypes were characteristic of the genus *Eimeria*, all including four sporocysts, each of which contained two sporozoites (Fig. 1). Oocysts were ovoid. Oocyst walls (OW) were ~1.2 µm (OTUy and z) or 1.8 µm (OTUz) thick and featured two layers; outer layer smooth and transparent (Table 2). OTUx possessed the largest oocysts of the three new species, with average length (L) 30.8 µm (28.2–32.8) and width (W) 23.8 µm (22.1–25.7), and an average L/W ratio of 1.29. OTUy was represented by oocysts of an intermediate size, L 26.7 µm (25.3–27.7), W 22.8 µm (21.5–23.9), and average L/W ratio 1.17. OTUz had the smallest oocysts of the three new species, L 17.7 µm (14.8–18.8), W 15.2 µm (13.2–16.8) and average L/W ratio 1.17. Micropyle (M) and oocyst residuum (OR) were absent, polar granules (PG) were present. Posterior and anterior refractile bodies (RB) and Stieda body (SB) were present. There was no sporocyst residuum (SR) (Table 2). Additional oocyst images are available in the Mendeley data repository at https://doi.org/10.17632/rtcympc7tm.1.

**Table 2:** Summary of sporulated oocyst characteristics for *Eimeria* Operational Taxonomic Units (OTUs) x, y and z.

Feature	OTUx	OTUy	OTUz
Oocyst shape	Ovoid	Ovoid	Ovoid
Oocyst wall: layers	2	2	2
Oocyst wall: surface	Smooth	Smooth	Smooth
Oocyst wall: thickness µm	~1.8	~1.2	~1.2
Oocyst length µm (range)	30.8 (28.2–32.8)	26.7 (25.3–27.7)	17.7 (14.8–18.8)
Oocyst width µm (range)	23.8 (22.1–25.7)	22.8 (21.5–23.9)	15.2 (13.2–16.8)
Oocyst length:width (range)	1.29 (1.19–1.38)	1.17 (1.16–1.20)	1.17 (1.06–1.27)
Micropyle (M)	No	No	No
Polar granule (PG)	Yes	Yes	Yes
Oocyst residuum (OR)	No	No	No
Sporocyst residuum (SR)	No	No	No
Stieda body (SB)	Yes	Yes	Yes

**Fig. 2:**
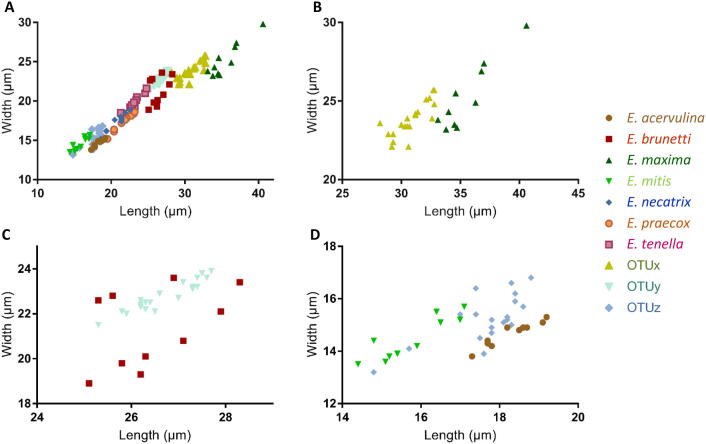
Dimensions of sporulated oocysts representing *Eimeria* collected from domestic chickens (*Gallus gallus domesticus*). (A) Oocysts from all seven *Eimeria* spp. that infect chickens and Operational Taxonomic Units (OTUs) x, y, and z. For close comparison subsets of (B) *Eimeria maxima* and OTUx, (C) *Eimeria brunetti* and OTUy, and (D) *Eimeria acervulina*, *Eimeria mitis*, and OTUz, are shown. Note: The scales used vary between panels to improve illustration of differences between oocysts with closely comparable dimensions.

For comparison, sporulated oocysts of the Houghton strains of *E. acervulina*, *E. brunetti*, *E. maxima*, *E. mitis*, *E. necatrix*, *E. praecox*, and *E. tenella* were also measured to complement published historic measurements (e.g. Long et al., 1976). Plotting sporulated oocyst dimensions by length and size revealed distinct but overlapping clusters for OTUs x, y, and z, most closely comparable to *E. maxima*, *E. brunetti*, and *E. acervulina*/*E. mitis*, respectively (Fig. 2), indicating that oocyst morphology alone is insufficient for differential diagnosis of OTU genotype parasites from the seven recognised species.

### 3.2 OTUs x and z present biological profiles that are distinct from the seven recognised *Eimeria* spp. that infect chickens

**Fig. 3:**
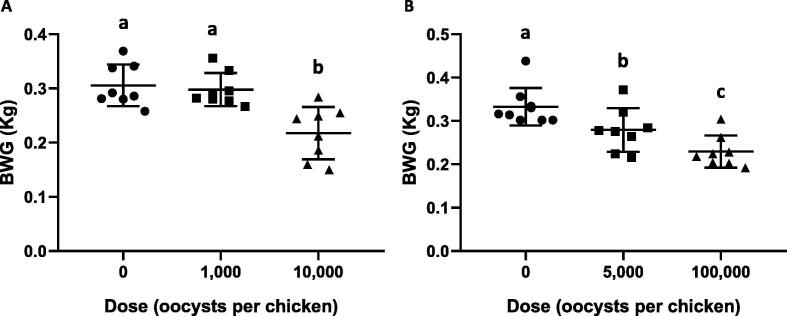
The effect of *Eimeria* Operational Taxonomic Unit (OTU) infection on broiler chicken bodyweight gain (BWG). Groups of eight 21 day old Ross 308 broiler chickens were infected by oral inoculation of (A) OTUx or (B) OTUz oocysts. Weight was measured at the time of infection and 10 days later. Data points indicate individual chickens, with the mean and S.D. presented for each group. Superscript letters identify groups that were statistically significantly different from each other within an experiment (*P* < 0.05). Note: The scales used on the Y axes vary between panels to improve visualisation of intra-group variation.

Experimental in vivo infection using OTUx identified a pre-patent period between 125 and 130 h, slightly longer than *E. maxima* and *E. brunetti*, the recognised species with the closest sporulated oocyst morphologies (Table 2, Fig. 2, Supplementary Table S2). Comparison of total OTUx oocyst production with that published for the seven recognised species using an equivalent dose (Bumstead and Millard, 1992) indicated an intermediate level of fecundity (Supplementary Table S2), although it should be stated that this was not a direct comparison and used different leghorn chicken breeds. Inoculation of 21-day old Ross 308 broiler-type chickens with 10,000 OTUx oocysts resulted in a 28.8% reduction in BWG compared with mock infected controls over the 10 days following challenge (*P* < 0.05; Fig. 3A). Inoculation of 1000 oocysts did not cause any significant change in BWG (2.5% reduction, not statistically significant). Combined, these results suggest an intermediate level of pathogenicity for OTUx (Table 3). Genotype-specific PCR targeting OTUx (primers as shown in Supplementary Table S1) was used to determine the range of parasite occurrence in intestinal samples collected from Lohmann Valo SPF chickens 112 h after infection. Samples collected from (1) the mid-point of the duodenum, (2) the distal end of the duodenum, (3) between the jejunum and ileum (around Meckel’s diverticulum), (4) the mid-point of the ileum, (5) the mid-point of one caecal pouch, and (6) the rectum were tested, indicating a range of replication for OTUx from the duodenum to the upper ileum (Table 3).

**Table 3:** Biological characteristics of *Eimeria* spp. and Operational Taxonomic Units (OTUs) that infect chickens (*Gallus gallus domesticus*). Details for the seven recognised species derived from Long et al., 1976, Eckert et al., 1995, where the darker shades indicate higher occurrence.

na, not available; SS, sexual stages.

^a^Information from Cantacessi et al. (2008); suggested location of replication.

A similar schedule to characterise OTUz parasites identified a pre-patent period between 130 and 135 h, notably longer than *E. acervulina* and *E. mitis*, the recognised species with the closest sporulated oocyst morphologies (Table 2, Fig. 2, Supplementary Table S2). Comparison of total OTUz oocyst production with that published for the seven recognised species using an equivalent dose indicated a low level of fecundity (Supplementary Table S2). Inoculation of 21-day old Ross 308 broiler chickens with 5000 OTUz oocysts resulted in a 16.1% reduction in BWG compared with mock infected controls over the 10 days following challenge (*P* < 0.05; Fig. 3B). Increasing the challenge dose to 100,000 oocysts reduced BWG further (31.1% compared with the control, *P* < 0.05; 17.9% compared with the lower dose challenge, *P* < 0.05). Combined, these results suggest a low level of pathogenicity (Table 3). Genotype-specific PCR targeting OTUz (primers as shown in Supplementary Table S2) revealed a range of replication from the duodenum to the upper ileum (Table 3).

A biological profile has not been developed here for OTUy in the absence of a viable, genetically pure stock. Previous reports suggest replication in the upper small intestinal tract and a pre-patent period of 132 h (Table 3; Cantacessi et al., 2008).

### 3.3 OTU isolates escape immune killing induced by commercial anticoccidial vaccines for chickens

Nigerian *Eimeria* field populations found by PCR to contain one or more OTU genotypes were passaged through triple vaccinated (Paracox® 8; MSD Animal Health, Milton Keynes, UK) chickens, supporting selective isolation of the OTU genotypes away from the seven recognised *Eimeria* spp. and preliminary assessment of vaccine escape (Table 1, Trial 1). It should be noted that Paracox® 8 contains attenuated isolates of all seven *Eimeria* spp. that are recognised to infect chickens, including two antigenically distinct *E. maxima* isolates to improve vaccine capacity (Williams and Gobbi, 2002). OTUs x and y were detected in all three starting populations (Ng1[1], 2[1] and 3[1], where [1] indicated first generation prior to selction), supplemented by OTUz in Ng2[1], and all were detected in the corresponding progeny populations after immune selection using genotype-specific PCR (Table 1). Replication of the live vaccine in previously vaccinated chickens was blocked, indicating apparently complete protection against homologous challenge. In vivo selection applied using triple Paracox® 8 vaccination prevented replication of the seven recognised *Eimeria* spp. in all but one example: Ng2[1], where a residual *E. acervulina* sup-population persisted in the progeny Ng2[2] population. Subsequent quantitative assessment of escape from triple Paracox® 8 vaccination by the selected Ng1[2] population, including OTUx and y sub-populations with no other species or OTU, confirmed that both continued to replicate, and that total oocyst production was not obviously compromised in vaccinated chickens (Table 1, Trial 2).

Following isolation of genetically pure OTUx and z populations from Nigeria by serial passage through vaccinated chickens, a more detailed assessment of escape from single heterologous (Paracox® 8) or homologous (self) vaccination was undertaken. It is important to note that the OTUx (Nig) and OTUz (Nig) populations used were isolated by serial selective passage, confirmed by species/OTU-specific PCR, and were not the progeny of single oocysts or sporocysts. Thus, each population was expected to retain some limited intra-specific genetic diversity. In the unvaccinated control group OTUx (Nig) was found to replicate well, producing ~13.8 million progeny oocysts per chicken (Table 1, Trial 3). Paracox® 8 vaccination failed to reduce OTUx (Nig) replication (7.2% lower, not statistically significant), but homologous vaccination using OTUx (Nig) induced apparently complete immune protection with no progeny oocysts detected. Similarly, OTUz (Nig) was found to replicate well in the unvaccinated control group, producing 30.2 million progeny oocysts per chicken (Table 1, Trial 3). No reduction in OTUz (Nig) oocyst shedding was observed in the heterologous Paracox® 8 vaccinated group (12.2% increase, not statistically significant), but again homologous vaccination using OTUz (Nig) induced apparently complete immune protection with no progeny oocysts detected.

Recognising that Paracox® 8 contains parasite lines isolated in Europe that may be antigenically heterologous to Australian or Nigerian isolates of the same species, we undertook an additional study of vaccine efficacy using the attenuated parasite lines included in HuveGuard® MMAT+NB and OTUz (Aus), all of which originated from Australia (Table 1, Trial 4). In the absence of vaccination, challenge using 20,000 OTUz (Aus) oocysts produced 5.2 million progeny oocysts per chicken. HuveGuard® vaccination reduced oocyst excretion to 4.4 million per chicken (15.6%). Homologous vaccination using OTUz (Aus) induced apparently complete immune protection with no progeny oocysts detected.

### 3.4 OTUs x, y, and z are genetically distinct from the seven recognised *Eimeria* spp. that infect chickens

**Fig. 4:**
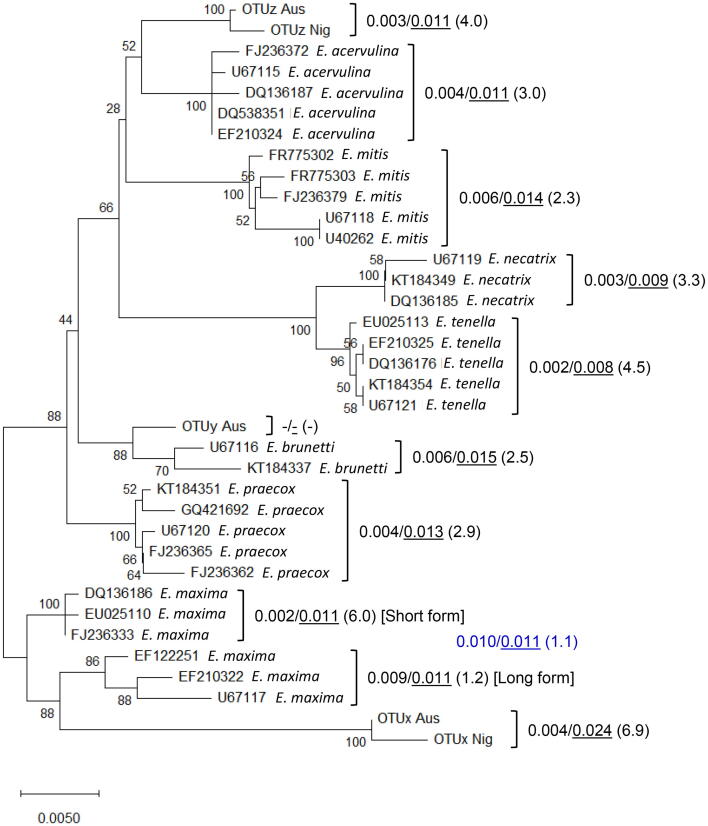
Optimal Neighbour-Joining tree inferred using a 1154 bp alignment of the partial *Eimeria* 18S rDNA locus. Evolutionary distances were calculated using the Kimura 2-parameter model. The number of base substitutions per site between sequences are shown to the right of the sequence identifiers, presented as a/b (x), where a = maximum number within a recognised species/Operational Taxonomic Unit (OTU) group, b = minimum between recognised species/OTUs, and the figure in parentheses is the fold difference. Pairwise analyses were conducted using the Maximum Composite Likelihood method. Figures shown in blue indicate analysis when the long and short form *Eimeria maxima* sequences were pooled. Aus, Australia; Nig, Nigeria.

Alignment of 18S rDNA sequences representing OTUs x, y, and z from Australia, OTUs x and z from Nigeria, and a panel of 31 published sequences representing the seven recognised *Eimeria* spp. from multiple studies and regions, resulted in a manually curated alignment of 1154 nucleotides. New sequences from Australian and Nigerian OTU parasites are available from the European Nucleotide Archive under accession number **PRJEB23613**. The ML, NJ, and MP methods produced comparable topology between species and OTU genotypes. OTUx was located on a branch with the long and short *E. maxima* 18S sequence forms (Fig. 4). The single OTUy sequence was most closely linked to *E. brunetti*, while the OTUz sequences were more distantly associated with *E. acervulina* and *E. mitis*. All associations for OTU parasites were consistent with the relatedness described for sporulated oocyst morphology. Intra-specific pairwise genetic distances were highest for *E. maxima*, *E. brunetti*, and *E. mitis* (0.006–0.009), rising to 0.010 when considering long and short *E. maxima* sequence types together. Inter-specific pairwise genetic distances where lowest for *E. tenella* and *E. necatrix* (0.008–0.009), demonstrating overlap between intra- and inter-specific results within the genus and suggesting that 18S rDNA was an inadequate genetic marker to assess the species status of OTU genotypes.

**Fig. 5:**
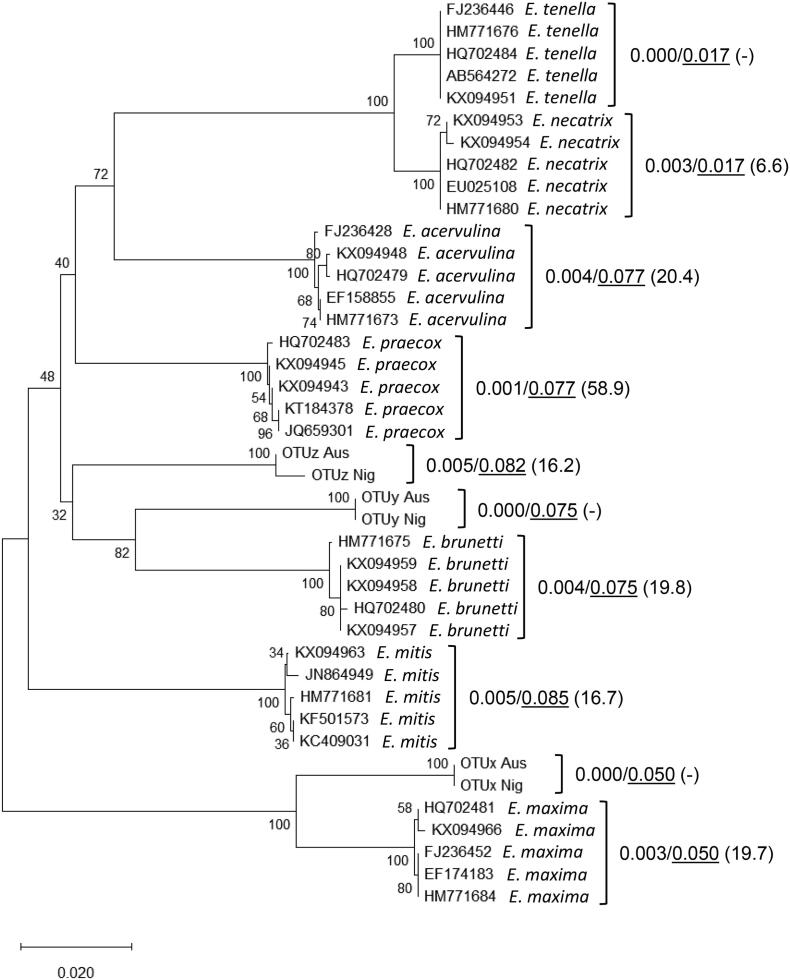
Optimal Neighbour-Joining tree inferred using a 791 bp alignment of the partial *Eimeria* cytochrome C oxidase I (COI) locus. Evolutionary distances were calculated using the Kimura 2-parameter model. The number of base substitutions per site between sequences are shown to the right of the sequence identifiers, presented as a/b (x), where a = maximum number within a recognised species/Operational Taxonomic Unit (OTU) group, b = minimum between recognised species/OTUs, and the figure in parentheses is the fold difference. Pairwise analyses were conducted using the Maximum Composite Likelihood method. Aus, Australia; Nig, Nigeria.

Comparison of partial mitochondrial COI gene sequences has previously been found to be more discerning than 18S rDNA sequences to differentiate *Eimeria* spp. (Ogedengbe et al., 2015). Alignment of partial COI sequences produced here representing OTUs x, y, and z from Australia and Nigeria with a panel of 35 published sequences representing the seven recognised *Eimeria* spp. resulted in a manually curated alignment of 791 nucleotides. New sequences from Australian and Nigerian OTU parasites are available from the European Nucleotide Archive under accession number **PRJEB23613**. Phylogenetic analysis using ML, NJ, and MP methods produced comparable topology between species and OTU genotypes. As for the 18S rDNA sequences, OTUx and y branched with *E. maxima* and *E. brunetti*, respectively (Fig. 5), both separated by greater evolutionary distances than the distinct species *E. necatrix* and *E. tenella*. Using the COI alignment OTUz, *E. acervulina*, and *E. mitis* were more distantly linked than suggested using the 18S rDNA alignment. Intra-specific pairwise genetic distances were highest for OTUz and *E. mitis* (0.005), 3.4-fold lower that the lowest inter-specific pairwise genetic distances calculated for *E. tenella* and *E. necatrix* (0.017).

**Fig. 6:**
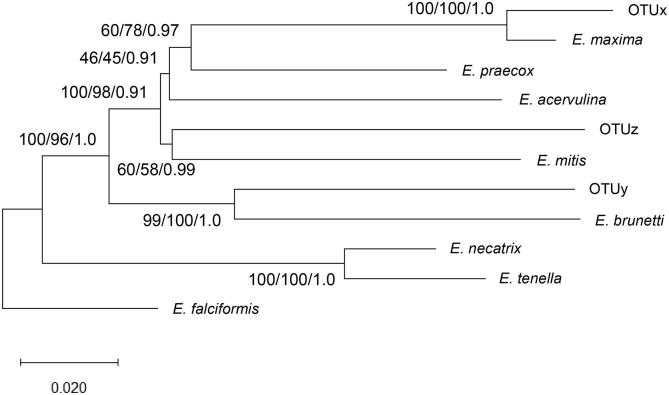
Optimal Neighbour-Joining tree inferred using a 4135 amino acid alignment of 56 concatenated *Eimeria* gene models rooted on *Eimeria falciformis*. Support for each node is presented, indicating outcomes from Maximum Likelihood (ML)/Neighbour-Joining (NJ)/Bayesian methods. For ML, the General Time Reversible (GTR) model was used with a gamma distribution. For NJ evolutionary distances were calculated using the Kimura 2-parameter model. For Bayesian inference analysis the GTR+G model was used, including four runs with 1,000,000 generations, a sample frequency of 10, and 25% burn-in.

Previous genetic studies of *Eimeria* OTU genomes have focused on the internal transcribed spacer (ITS) repeat, within the nuclear genome, and sequences from the mitochondrial genome (Cantacessi et al., 2008, Clark et al., 2016, Morgan and Godwin, 2017). Here, phylogenetic analysis using a fragment of the mitochondrial COI successfully resolved the *Eimeria* spp. and OTU genotypes, although comparison with the 18S rDNA phylogeny revealed some inconsistencies. To generate new resources and support improved genetic characterisation we have undertaken limited genome sequencing using Illumina MiSeq with single Nextera XT libraries for OTUs x, y, and z, assembling preliminary genome sequence assemblies and predicting candidate coding sequences. The raw sequences and associated assemblies have been deposited in the European Nucleotide Archive under the study accession number **PRJEB40060**, with the sample accession numbers **ERS5037937-9**. The draft genome assemblies include 42.9 Mb (OTUx), 58.0 Mb (OTUy) and 50.6 Mb (OTUz) nucleotides, represented by 14–27× sequence coverage assembled into contigs >500 bp with GC contents between 48.5 and 49.8% (Supplementary Table S3). Panels of 10,309, 12,777, and 11,705 gene models have been predicted from the OTUx, y, and z genomes, respectively. Comparison of OTU gene models with *E. acervulina*, *E. brunetti*, *E. maxima*, *E. mitis*, *E. necatrix*, *E. praecox*, and *E. tenella*, together with *E. falciformis* as an outgroup, identified 56 orthologue groups that were suitable for further analysis. ML, NJ, and Bayesian inference analysis of a concatenated amino acid alignment representing all 56 orthologue groups supported the links between OTUx and y with *E. maxima* and *E. brunetti*, respectively, highlighting equivalent or greater evolutionary distances than separate *E. necatrix* and *E. tenella* (Fig. 6). The concatenated amino acid alignment used is available from the Mendeley data repository under the accession https://doi.org/10.17632/nphkwt6h3j.1. OTUz presented the greatest evolutionary divergence from the recognised species, but support from ML and NJ was relatively low.

## 4 Discussion

Coccidian parasites that infect chickens were first described more than a century ago (Chapman, 2003), with descriptions of at least nine candidate species resolved to seven recognised *Eimeria* spp. (Gasser et al., 2005, Vrba et al., 2011). The *status quo* was disrupted in 2007 with a report describing three cryptic OTUs defined by novel ITS-2 amplicon electrophoresis profiles (Morris et al., 2007). Subsequent locus-specific PCR and amplicon sequencing reiterated their cryptic status (Cantacessi et al., 2008, Morgan and Godwin, 2017), while molecular surveillance suggested an unexpectedly polarised global distribution (Clark et al., 2016). Recent reports describing detection of all three OTU genotypes in the United States suggest a far wider prevalence (Hauck et al., 2019), prompting renewed interest in their status.

**Fig. 7:**
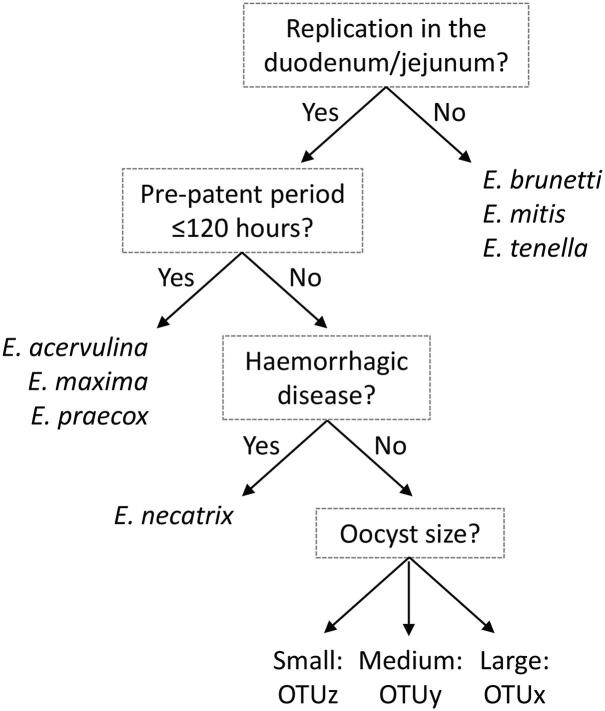
Dichotomous key discriminating Operational Taxonomic Units (OTUs) x, y and z from the seven recognised *Eimeria* spp. that infect chickens.

Discrimination between distinct *Eimeria* spp. is commonly challenging, often relying on subjective assessment of sporulated oocyst morphology and pathological features such as site of replication and the presence or absence of characteristic intestinal lesions. To assess the species status of OTUs x, y, and z we have defined a series of biological parameters commonly used to speciate *Eimeria*, including oocyst shape (length, width, and length:width ratio) (Bandoni and Duszynski, 1988), pre-patent period, and intestinal site(s) of replication. Oocyst size and shape is the most accessible species characteristic. Here, isolates of all three OTU genotypes displayed oocyst morphologies that overlapped with one (OTUs x and y) or two (OTUz) recognised *Eimeria* spp. (Fig. 2). Oocysts of OTUx were found on average to be widest of the species and genotypes that infect chickens, but the difference was not consistent in all examples (Supplementary Table S2). However, all three OTUs displayed longer pre-patent periods than reported for the recognised species with the closest oocyst morphology, all above 120 h, only exceeded by *E. necatrix* and *E. tenella* (Table 3). It has previously been suggested that all three OTU genotypes replicate in the upper small intestine (Cantacessi et al., 2008), confirmed here for OTUs x and z, permitting differentiation from *E. brunetti*, *E. mitis*, and *E. tenella* (Table 3). Combined, these features permit construction of a dichotomous key to differentiate OTU genotypes from each other and the seven recognised *Eimeria* spp. that infect domestic chickens (Fig. 7).

Attempting to define the provenance of *Eimeria* OTUs x, y, and z identifies obvious parallels with the dubious species *E. hagani* and *E. mivati*. Published descriptions are most widely available for the latter, suggesting an average sporulated oocyst size of 15.6 µm (range 10.7–20.0) × 13.4 µm (10.1–15.3), replication along the full length of the intestinal tract, and a pre-patent period of 93 h (Edgar and Seibold, 1964). *Eimeria hagani* has been less well defined, but its sporulated oocysts have been reported to measure 19.1 µm (15.8–20.9) × 17.6 µm (14.3–19.5), with replication in the duodenum and a pre-patent period of 99 h (Reid, 1968). Neither of these profiles match with OTUs x, y, or z, and we conclude that these two species remain *nomina dubia*, despite their registered presence in live anticoccidial vaccines such as Coccivac-D® (MSD Animal Health).

*Eimeria* spp. can also be defined by the specificity of the immune response induced by natural infection. Immunity to each *Eimeria* sp. that infects the chicken is usually absolutely parasite species-specific, with no significant cross-protection against subsequent challenge by heterologous species (McDonald and Shirley, 2009). Here, a multiple live anticoccidial vaccination approach was applied using Paracox® 8 or HuveGuard® MMAT+NB to selectively isolate OTU parasites away from recognised *Eimeria* spp. during in vivo passage of complex field and candidate pure OTU populations. Recovery of OTU oocysts demonstrated escape from immune killing, indicating significant antigenic diversity. Quantitative studies assessing escape by OTUs x and z from immunity induced by prior infection with live commercial vaccines (heterologous) or self (homologous) confirmed the absence of cross-protective immunity. Most noteworthy was the escape of an Australian OTUz isolate from vaccination using the Australian parasite lines included in HuveGuard® MMAT+NB. While partial strain-specific immunity has been described following infection with *E. acervulina* (Joyner, 1969), *E. mitis* (McDonald et al., 1985), *E. maxima* (Smith et al., 2002), or *E. tenella* (Awad et al., 2013), indicating that antigenic type alone cannot confirm identification of a distinct species, the results do support the biological characterisation of each OTU.

Genetic characterisation offers a powerful approach to define relatedness between organisms. For *Eimeria*, studies have primarily been restricted to a small panel of nuclear and mitochondrial loci including the 18S rDNA, ITS sequences 1 and 2, and COI (Ogedengbe et al., 2015). Application of these tools has supported studies including resolution of the taxonomic status of *E. mivati* and exploration of evolutionary relatedness among *Eimeria* derived from different galliform birds (Miska et al., 2010, Vrba et al., 2011). Phylogenetic comparison of partial 18S rDNA sequences supported biological characterisation of OTU parasites, identifying close links between each OTU and the *Eimeria* sp. with the most closely comparable oocyst morphology (Fig. 2, Fig. 4). However, overlapping intra- and inter-specific diversity suggested that 18S rDNA sequences were insufficient to provide a robust assessment of true species status, as described by others when assessing parasites derived from closely related host species (Jarquin-Diaz et al., 2020). Comparison of partial COI sequences proved more effective, confirming relatedness between OTUx and *E. maxima*, as well as OTUy and *E. brunetti*, in agreement with previous analysis of *Eimeria* spp. and OTU whole mitochondrial genomes (Morgan and Godwin, 2017). COI has been similarly discriminatory in some, but not all studies of *Eimeria*, including examples from closely related rodent hosts (Jarquin-Diaz et al., 2019; Jarquin-Diaz et al., 2020). Interestingly, while the location of OTUz has been less stable between phylogenies, it has consistently appeared more distant from the recognised species than the other OTUs. To resolve this instability, and to provide resources for future investigation, we generated low coverage genome sequence datasets for each OTU. Using Illumina MiSeq with Nextera XT libraries permitted data generation with limited DNA input, at the cost of highly fragmented and likely incomplete sequence assemblies (Supplementary Table S3). Prediction of putative coding sequences within each OTU genome sequence assembly supported preparation of 56 gene-specific amino acid alignments from the seven recognised *Eimeria* spp. that infect chickens and all three OTUs, adding *E. falciformis* as an outgroup. Bayesian inference of the concatenated amino acid sequence phylogeny provided the strongest genetic evidence of the relationships between all 11 parasites, supported by ML and NJ estimations (Fig. 6). Genetic distances between each OTU and its closest relative equivalent to, or greater than that between the separate species *E. tenella* and *E. necatrix* support the annotation of each OTU as a distinct species.

Combined, the biological and genetic characterisation described here support designation of OTUs x, y, and z as new *Eimeria* spp., with evidence strongest for the latter. Further evidence may be considered in future studies, for example assessing the ability of each OTU to hybridise (cross-fertilise) with its closest biological or genetic relative (the biological species concept). Long considered a stringent threshold for species-level definition, the ability to produce viable hybrid progeny has been used to discriminate between species and sub-species/strain variants. However, it is worth noting that hybridisation between species has been described for other apicomplexans such as *Plasmodium berghei* and *Plasmodium yoelii* (Ramiro et al., 2015), suggesting that such a concept may not be helpful for apicomplexans. Acknowledging the long history of mis-establishing new *Eimeria* spp. that infect poultry, we tentatively suggest the name *Eimeria lata* n. sp. for OTUx, highlighting the widest average oocyst width of any *Eimeria* sp. that infects chickens (Supplementary Table S2; registered with ZooBank under the identifier 4A140B25-9C29-4C9C-BEB5-E48A180E0EC0). We suggest *Eimeria nagambie* n. sp. for OTUy (ZooBank 907847E3-CE39-4367-B2FF-9A8BA187C304), and *Eimeria zaria* n. sp. for OTUz (ZooBank 733D2D34-031F-418E-9C6C-76CA2012CA3F), reflecting the Australian and Nigerian origins of the isolates used to describe the species.

Beyond the primarily academic exercise that is determination of species status, a question more relevant to chicken production and food security relates to the risk posed by *Eimeria* OTU parasites. More than 68 billion chickens and 1.3 trillion eggs were produced in the World in 2018 (FAOSTAT, http://www.fao.org/faostat/), representing a major contribution to human nutrition and economic prosperity. As producers strive to reduce drug use in livestock production, many are turning to live anticoccidial vaccines to control *Eimeria* and prevent coccidiosis (Chapman and Jeffers, 2014). It is important to highlight that at least two market leading anticoccidial vaccines do not appear to protect against OTU genotype *Eimeria*. The occurrence of OTUs x and y have previously been associated with persistent coccidiosis problems on an Australian broiler breeder farm in vaccinated stock (Morris et al., 2007). The presence or absence of OTUs x and z has also been linked to the economic success of broiler and layer enterprises in Ghana, Tanzania, and Zambia (Fornace et al., 2013). Here, we have found that OUT x or z infection significantly compromised BWG by Ross 308 broiler chickens, one of the most common commercial lines globally (Havenstein et al., 2003), producing a malabsorptive condition likely to incur economic and welfare consequences. Increasing the use of anticoccidial vaccines that do not protect against OTU species of *Eimeria* is likely to encourage higher levels of occurrence and, in the absence of alternative controls, disease. In response, existing live anticoccidial vaccines may require modification to include one or more OTU parasite lines, at least for more valuable, longer-lived layer and breeder stock.

The origin of the *Eimeria* OTU parasites described here remains unclear. While it is possible that OTUs x, y, and z are emerging new pathogens, it is more likely that they represent minority *Eimeria* sub-populations that have previously been undetected. Their close resemblance to other *Eimeria* spp. and lack of distinct pathognomonic signature may have precluded their detection by traditional microscopic and pathological approaches. Similarly, the narrow range of molecular assays developed for *Eimeria* may have provided insufficient sensitivity and/or specificity to identify them. Alternatively, the three OTU genotypes may have originated from one or more other host species, for example junglefowl or other closely related Galliformes. The seven established *Eimeria* spp. that infect chickens appear to be host species-specific, but species from some other gallinaceous birds have proved capable of adaptation to foreign hosts (Vrba and Pakandl, 2015). Changes in husbandry practises, including increased use of anticoccidial vaccination and larger numbers of backyard poultry, may now provide opportunities for minority *Eimeria* sub-populations to proliferate. Indeed, deeper sampling of *Eimeria* populations using next generation sequencing protocols may support detection of additional *Eimeria* OTUs in the future (Hinsu et al., 2018, Hauck et al., 2019). In a recent study, Hauck and colleagues described detection of OTUs x, y, and z in backyard but not commercial broiler chickens in the United States, supplemented by a panel of new sequence types (Hauck et al., 2019). Such descriptions are relevant, although detection of *Eimeria* sequences associated with non-chicken hosts including ferrets, rodents, and rock partridge in chicken faecal samples may indicate environmental contamination with non-replicating *Eimeria* oocysts or DNA from other sources, possibly influenced by chicken foraging behaviour, rather than active infections (Hinsu et al., 2018).

To conclude, three cryptic *Eimeria* OTUs detected circulating in chickens possess sufficient genetic and biological diversity to be considered as new species. The ability of these parasites to compromise chicken growth and escape immunity induced by commercial anticoccidial vaccines indicates a notable threat to chicken health, welfare, and productivity. We propose the names *E. lata* n. sp., *E. nagambie* n. sp. and *E. zaria* n. sp. for these new species, prompting a requirement for existing *Eimeria* diagnostics and controls to be re-assessed.

Taxonomic summaries

*Eimeria lata* n sp. (previously OTUx)

Description: sporulated oocysts assessed after recovery from faeces and sporulation under ambient conditions for a minimum of 48 h measure 30.8 (range 28.2–32.8) × 23.8 (22.1–25.7) µm with a length:width ratio of 1.29 (1.19–1.38). Smooth, ovoid oocyst with wall ~1.8 µm thick. Stieda body and polar granule present. Micropyle, oocyst residuum and sporocyst residuum absent.

Type host: domestic chicken (*Gallus gallus domestics*).

Other hosts: not known.

Type locality: Australia, Nigeria.

Other localities: Ghana, India, Tanzania, Uganda, Unites States of America, Venezuela (Clark et al., 2016, Hauck et al., 2019).

Location in host: duodenum, jejunum.

Prepatent period: 125–130 h.

Patent period: not known.

Pathogenicity studies: experimental infection results in reduced weight gain, blood visible in faeces (haemorrhagic) at higher doses.

Diagnosis: detection of oocysts in faeces morphologically similar to *E. maxima*. Differentiate by PCR as described here or following the dichotomous key (Fig. 7).

Material deposited: phototype, purified oocysts, frozen sporocysts and genomic DNA at the Royal Veterinary College, UK. Raw genome sequence reads, associated assembly and gene predictions are available at GenBank under the accession number ERS5037937 (Australian isolate). Gene models used for phylogenetic comparison are available at the Mendeley data repository under the accession https://doi.org/10.17632/nphkwt6h3j.1. Specific sequences are available for the 18S rDNA (GenBank accession number LT964972) and COI (LR990836, LR877716) loci, in addition to the complete mitochondrial genome published by others (KX094967; (Morgan and Godwin, 2017)).

Etymology: this species has been named in recognition of the widest average oocyst width of any *Eimeria* species found to date to infect domestic chickens, using *lata* to indicate ‘wide’.

*Eimeria nagambie* n sp. (previously OTUy)

Description: sporulated oocysts assessed after recovery from faeces and sporulation under ambient conditions for a minimum of 48 h measure 26.7 (25.3–27.7) × 22.8 (21.5–23.9) µm with a length:width ratio of 1.17 (1.16–1.20). Smooth, ovoid oocyst with wall ~1.2 µm thick. Stieda body and polar granule present. Micropyle, oocyst residuum and sporocyst residuum absent.

Type host: domestic chicken (*Gallus domestics*).

Other hosts: not known.

Type locality: Australia.

Other localities: Unites States of America (Clark et al., 2016, Hauck et al., 2019).

Location in host: duodenum, jejunum.

Prepatent period: 132 h.

Patent period: not known.

Pathogenicity studies: not available.

Diagnosis: detection of oocysts in faeces morphologically similar to *E. brunetti*. Differentiate by PCR as described here or following the dichotomous key (Fig. 7).

Material deposited: phototype and genomic DNA at the Royal Veterinary College, UK. Raw genome sequence reads, associated assembly and gene predictions are available at GenBank under the accession number ERS5037938 (Australian isolate). Gene models used for phylogenetic comparison are available at the Mendeley data repository under the accession https://doi.org/10.17632/nphkwt6h3j.1. Specific sequences are available for the 18S rDNA (GenBank accession number LT964973) and COI (LR877717, LR877718) loci, in addition to the complete mitochondrial genome published by others (KX094960; (Morgan and Godwin, 2017)).

Etymology: this species has been named in recognition of the location of the first sequenced and phenotyped isolate, collected in Nagambie, Victoria, Australia.

*Eimeria zaria* n sp. (previously OTUz)

Description: sporulated oocysts assessed after recovery from faeces and sporulation under ambient conditions for a minimum of 48 h measure 17.7 (14.8–18.8) × 15.2 (13.2–16.8) µm with a length:width ratio of 1.17 (1.06–1.27). Smooth, ovoid oocyst with wall ~1.2 µm thick. Stieda body and polar granule present. Micropyle, oocyst residuum and sporocyst residuum absent.

Type host: domestic chicken (*Gallus gallus domestics*).

Other hosts: not known.

Type locality: Australia, Nigeria.

Other localities: Ghana, India, Tanzania, Uganda, Unites States of America, Venezuela, Zambia (Clark et al., 2016, Hauck et al., 2019).

Location in host: duodenum, jejunum.

Prepatent period: 130–135 h.

Patent period: not known.

Pathogenicity studies: experimental infection results in reduced weight gain.

Diagnosis: detection of oocysts in faeces morphologically similar to *E. acervulina* and *E. mitis*. Differentiate by PCR as described here or following the dichotomous key (Fig. 7).

Material deposited: phototype, purified oocysts, frozen sporocysts and genomic DNA at the Royal Veterinary College, UK. Raw genome sequence reads, associated assembly and gene predictions are available at GenBank under the accession number ERS5037939 (Australian isolate). Gene models used for phylogenetic comparison are available at the Mendeley data repository under the accession https://doi.org/10.17632/nphkwt6h3j.1. Specific sequences are available for the 18S rDNA (GenBank accession number LT964974, LR877466) and COI (HG992976, LR877720) loci, in addition to the complete mitochondrial genome published by others (KX094955, KX094956; (Morgan and Godwin, 2017)).

Etymology: this species has been named in recognition of the location of the phenotyped isolate, collected in Zaria, Kaduna state, Nigeria.
