# Supplementary material for: Genetic and biological characterisation of three cryptic Eimeria operational taxonomic units that infect chickens (Gallus gallus domesticus)
Source: Int J Parasitol. 2021 Jul;51(8):621–34. doi: 10.1016/j.ijpara.2020.12.004 (PMC8186487; doi:10.1016/j.ijpara.2020.12.004)
Supplement: Supplementary data 1 [file mmc1.docx]

**Supplementary Table S1**. PCR primers used for molecular identification of *Eimeria* spp. and Operational Taxonomic Unit (OTU) genotypes.

| Target | Primer | Sequence (5' - 3') | Annealing (^o^C) | Amplicon size (bp) | Reference |
| --- | --- | --- | --- | --- | --- |
| *Eimeria acervulina* | ACE-F | GCAGTCCGATGAAAGGTATTTG | 56 | 103 | Vrba et al. (2010) |
|  | ACE-R | GAAGCGAAATGTTAGGCCATCT |  |  | Vrba et al. (2010) |
| *Eimeria brunetti* | BRU-F | AGCGTGTAATCTGCTTTTGGAA | 56 | 118 | Vrba et al. (2010) |
|  | BRU-R | TGGTCGCAGACGTATATTAGGG |  |  | Vrba et al. (2010) |
| *Eimeria maxima* | MAX-F | TCGTTGCATTCGACAGATTC | 56 | 138 | Vrba et al. (2010) |
|  | MAX-R | TAGCGACTGCTCAAGGGTTT |  |  | Vrba et al. (2010) |
| *Eimeria mitis* | MIT-F | CAAGGGGATGCATGGAATATAA | 56 | 115 | Vrba et al. (2010) |
|  | MIT-R | CAAGACGAATGGAATCAATCTG |  |  | Vrba et al. (2010) |
| *Eimeria necatrix* | NEC-F | AACGCCGGTATGCCTCGTCG | 56 | 134 | Vrba et al. (2010) |
|  | NEC-R | GTACTGGTGCCAACGGAGA |  |  | Vrba et al. (2010) |
| *Eimeria praecox* | PRA-F | CACATCCAATGCGATATAGGG | 56 | 117 | Vrba et al. (2010) |
|  | PRA-R | ACAGAAAAACGCAAAGAGCAA |  |  | Vrba et al. (2010) |
| *Eimeria tenella* | TEN-F | TCGTCTTTGGCTGGCTATTC | 56 | 100 | Vrba et al. (2010) |
|  | TEN-R | CAGAGAGTCGCCGTCACAGT |  |  | Vrba et al. (2010) |
|  |  |  |  |  |  |
| OTUx | OTU-Xf2 | GGGTAGAGCCAGGGGTAGAG | 58 | 1,018 | This study |
|  | OTU-Xr2 | CGTAGTCCCAAGTGCCAACT |  |  | This study |
| OTUy | OTU-Yf1 | CAAGAAGTACACTACCACAGCATG | 56 | 346 | Fornace et al. (2013) |
|  | OTU-Yr1 | ACTGATTTCAGGTCTAAAACGAAT |  |  | Fornace et al. (2013) |
| OTUz | OTU-Zf1 | TATAGTTTCTTTTGCGCGTTGC | 58 | 147 | Fornace et al. (2013) |
|  | OTU-Zr1 | CATATCTCTTTCATGAACGAAAGG |  |  | Fornace et al. (2013) |

**References**

Fornace, K.M., Clark, E.L., Macdonald, S.E., Namangala, B., Karimuribo, E., Awuni, J.A., Thieme, O., Blake, D.P., Rushton, J., 2013. Occurrence of *Eimeria* species parasites on small-scale commercial chicken farms in Africa and indication of economic profitability. PLoS ONE 8, e84254.

Vrba, V., Blake, D.P., Poplstein, M., 2010. Quantitative real-time PCR assays for detection and quantification of all seven *Eimeria* species that infect the chicken. Vet Parasitol 174, 183-190.

**Supplementary Table S2.** Comparison of *Eimeria* spp. size, replication and fecundity. Oocyst dimensions for the seven recognised species as stated by Long et al. (1976). Data for replication and fecundity of the seven recognised species that infect chickens derived from Bumstead and Millard (1992).

| *Eimeria* spp. | Oocyst length × width (µm) | Strain | Host breed | Dose (oocysts per bird) | Yield (oocysts per bird) | Reference | Actual fecundity | Relative fecundity |
| --- | --- | --- | --- | --- | --- | --- | --- | --- |
| *E. acervulina* | 18.3 × 14.6 | Houghton | Brown Leghorn | 100 | 7.27E+07 |  | 727,000 | +++++ |
| *E. brunetti* | 24.6 × 18.8 | Houghton | Brown Leghorn | 100 | 2.42E+07 |  | 242,000 | +++ |
| *E. maxima* | 30.5 × 20.7 | Watton | Brown Leghorn | 100 | 1.83E+07 | Bumstead | 183,000 | ++ |
| *E. mitis* | 15.6 × 14.2 | Houghton | Brown Leghorn | 100 | 4.55E+07 | and Millard | 455,000 | ++++ |
| *E. necatrix* | 20.4 × 17.2 | Houghton | Brown Leghorn | 100 | 7.13E+06 | (1992) | 71,300 | + |
| *E. praecox* | 21.3 × 17.1 | Houghton | Brown Leghorn | 100 | 6.44E+07 |  | 644,000 | +++++ |
| *E. tenella* | 22.0 × 19.0 | Weybridge | Brown Leghorn | 100 | 1.83E+07 |  | 183,000 | ++ |
| OTUx | 30.8 × 23.8 | Nagambie | Lohmann Valo | 100 | 1.22E+07 | This study | 122,000 | ++ |
| OTUy | 26.7 × 22.8 | Nagambie |  | na | na |  | na | na |
| OTUz | 17.7 × 15.2 | Nagambie | Lohmann Valo | 100 | 2.26E+07 | This study | 226,000 | +++ |

na, not available; OTU, Operational Taxonomic Unit.

**Reference**

Bumstead, N., Millard, B., 1992. Variation in susceptibility of inbred lines of chickens to seven species of *Eimeria*. Parasitology 104, 407-413.

**Supplementary Table S3.** Genome assembly statistics for the *Eimeria* Operational Taxonomic Units (OTUs) isolated from domestic chickens (*Gallus gallus domesticus*).

|  | OTUx | OTUy | OTUz |
| --- | --- | --- | --- |
| Genome assembly size (Mb) | 42.9 | 58 | 50.6 |
| ~fold coverage (contigs >500 bp) | 24 | 14 | 27 |
| GC (%) | 48.5 | 49.8 | 48.7 |
| No. contigs (>0 bp) | 16,072 | 40,800 | 27,925 |
| No. contigs (>5,000 bp) | 1,775 | 1,658 | 2,043 |
| Largest contig (bp) | 199,092 | 83,507 | 141,671 |
| N50 | 10,047 | 2,900 | 5,499 |
| Gene models (N) | 10,309 | 12,777 | 11,705 |
